# The prognostic impact of insulin resistance surrogates in patients with acute myocardial infarction with and without type 2 diabetes

**DOI:** 10.1186/s12933-024-02240-z

**Published:** 2024-04-29

**Authors:** Dominika Rokicka, Bartosz Hudzik, Marta Wróbel, Tomasz Stołtny, Dorota Stołtny, Alicja Nowowiejska-Wiewióra, Sonia Rokicka, Mariusz Gąsior, Krzysztof Strojek

**Affiliations:** 1 Department of Internal Medicine, Diabetology and Cardiometabolic Disorders, Faculty of Medical Sciences Zabrze , Medical University of Silesia, Katowice, ul. M. Curie-Skłodowskiej 9, 41-800 Zabrze, Poland; 2 Third Department of Cardiology Faculty of Medical Sciences Zabrze, Medical University of Silesia, Katowice, ul. M. Curie-Skłodowskiej 9, 41-800 Zabrze, Poland; 3 Department of Cardiovascular Disease Prevention in Bytom, Medical University of Silesia, Katowice, Poland; 4 District Hospital of Orthopaedics and Trauma Surgery Piekary Śląskie, ul. Bytomska 62, 41-940 Piekary Śląskie, Poland; 5 Medical University of Gdańsk, ul. Marii Skłodowskiej-Curie 3a, 80-210 Gdańsk, Poland

**Keywords:** STEMI, NSTEMI, Type 2 diabetes mellitus, TyG index, Tyg-BMI, METS-IR, In-hospital death, MACCE

## Abstract

**Background:**

Cardiovascular disease is the major cause of morbidity and mortality, particularly in type 2 diabetes mellitus (T2DM). Novel markers of insulin resistance and progression of atherosclerosis include the triglycerides and glucose index (TyG index), the triglycerides and body mass index (Tyg-BMI) and the metabolic score for insulin resistance (METS-IR). Establishing independent risk factors for in-hospital death and major adverse cardiac and cerebrovascular events (MACCE) in patients with myocardial infarction (MI) remains critical. The aim of the study was to assess the risk of in-hospital death and MACCE within 12 months after ST-segment elevation myocardial infarction (STEMI) and non-ST-segment elevation myocardial infarction (NSTEMI) in patients with and without T2DM based on TyG index, Tyg-BMI and METS-IR.

**Methods:**

Retrospective analysis included 1706 patients with STEMI and NSTEMI hospitalized between 2013 and 2021. We analyzed prognostic value of TyG index, Tyg-BMI and METS-IR for in-hospital death and MACCE as its components (death from any cause, MI, stroke, revascularization) within 12 months after STEMI or NSTEMI in patients with and without T2DM.

**Results:**

Of 1706 patients, 58 in-hospital deaths were reported (29 patients [4.3%] in the group with T2DM and 29 patients [2.8%] in the group without T2DM; p = 0.1). MACCE occurred in 18.9% of the total study population (25.8% in the group with T2DM and 14.4% in the group without T2DM; p < 0.001). TyG index, Tyg-BMI and METS-IR were significantly higher in the group of patients with T2DM compared to those without T2DM (p < 0.001). Long-term MACCE were more prevalent in patients with T2DM (p < 0.001). The area under the ROC curve (AUC-ROC) for the prediction of in-hospital death and the TyG index was 0.69 (p < 0.001). The ROC curve for predicting in-hospital death based on METS-IR was 0.682 (p < 0.001). The AUC-ROC values for MACCE prediction based on the TyG index and METS-IR were 0.582 (p < 0.001) and 0.57 (p < 0.001), respectively.

**Conclusions:**

TyG index was an independent risk factor for in-hospital death in patients with STEMI or NSTEMI. TyG index, TyG-BMI and METS-IR were not independent risk factors for MACCE at 12 month follow-up. TyG index and METS-IR have low predictive value in predicting MACCE within 12 months after STEMI and NSTEMI.

## Introduction

Cardiovascular complications are a major cause of morbidity and mortality, particularly in type 2 diabetes mellitus (T2DM) [1]. Myocardial infarction (MI), results from myocardial ischemia due to a disproportion between myocardial oxygen supply and demand [2]. Atherosclerosis is the most common cause of MI. Risk factors for coronary artery atherosclerosis and its progression have been identified, which allowed the implementation of preventive and therapeutic measures to reduce its prevalence and improve prognosis [3]. Major adverse cardiovascular and cerebrovascular events (MACCE) are still the cause of frequent hospitalizations and death, particularly in developing countries [4]. Patients with T2DM are at high or even very high risk of cardiovascular disease (CVD). The occurrence of further cardiovascular incidents in this group of patients is much more common than in the general population [5]. Therefore, improvement in risk assessment of in-hospital death and MACCE defined as death from any cause, MI, stroke and repeat revascularization during long-term follow-up for the general population and T2DM population has significant implications in managing patients with MI.

Many studies have confirmed the impact of insulin resistance and lipid and glucose metabolism dysregulation on the progression of coronary artery disease (CAD). Studies have demonstrated that insulin resistance, which is the main pathogenetic mechanism in the development of T2DM, is a factor influencing the progression of macrovascular complications in this group of patients and a factor in the development of CVD in patients without T2DM [3, 6–8]. Many methods have been developed to estimate insulin resistance. The most commonly used being the Homeostasis Model Insulin-Resistance Assessment (HOMA-IR). The distribution of HOMA-IR varies according to demographic characteristics of patients, such as age, gender and race, which makes it difficult to estimate the optimal cut-off point [9].

Therefore, the search continues for simpler and reliable indicators that could estimate insulin resistance and the associated progression of atherosclerosis without using insulin assays. TyG index (triglycerides and glucose index), Tyg-BMI (triglycerides and body mass index) and METS-IR (metabolic score for insulin resistance), are indices that incorporate basic laboratory parameters, such as fasting serum glucose, triglycerides, HDL-cholesterol, and anthropometric measurements, including weight and height [3, 6]. These formulas seem to be reliable indices of insulin resistance and independent predictors of atherosclerosis progression in the general population and T2DM patients [3, 6, 7]. Estimating reliable indicators of in-hospital death and MACCE in patients after acute coronary syndrome (ACS) remains a critical issue.

We set out to investigate prognostic values of TyG index, Tyg-BMI and METS-IR for the occurrence of in-hospital death and MACCE within 12 months after ST-segment elevation myocardial infarction (STEMI) or non-ST-segment elevation myocardial infarction (NSTEMI).

## Materials and methods

The study conforms to the Declaration of Helsinki. Informed consent for data analysis was obtained from the patients according to the Polish law on patients rights regarding data registration. Approval for analyzing recorded data was waived by the local bioethics committee on human research given the retrospective nature of the study.

This retrospective analysis included 1706 patients hospitalized for STEMI and NSTEMI between 2013 and 2021. Detailed anthropometric data were routinely collected from patients during hospitalization. Additionally, biochemical tests and percutaneous coronary intervention (PCI) were performed.

The study population was divided into two groups:

- Group 1—patients with T2DM (n = 680)
- Group 2- patients without T2DM (n = 1026).

The inclusion criterion was STEMI or NSTEMI in patients who underwent coronary angiography. STEMI was diagnosed when clinical symptoms of myocardial ischemia were accompanied by an increase in cardiac troponin levels (myocardial necrosis marker) at least one value above the 99th percentile upper reference limit with ST-segment changes in the electrocardiogram (ECG) (i.e., ST-segment elevation at the J point in V2 and V3 leads of ≥ 2 mm in men aged ≥ 40 years and of ≥ 2.5 mm in men < 40 years of age and of ≥ 1.5 mm in women and/or ST-segment elevation of ≥ 1 mm in ≥ 2 contiguous electrocardiographic leads or the presence of a new left bundle branch block [10].

In turn, NSTEMI was diagnosed when clinical symptoms of myocardial ischemia were accompanied by an increase in cardiac troponin levels with at least one value above the 99th percentile upper reference limit without persistent ST-segment elevation on ECG and the presence of ST-segment depression of ≥ 0.5 mm or the presence of a negative T-wave or reversal of previously negative T-waves to positive [2, 11].

The exclusion criteria were eligibility for coronary artery bypass grafting (CABG) after coronary angiography, patients with type 1 diabetes mellitus and diabetes mellitus of other etiologies.

Clinical, laboratory, echocardiographic, and angiographic parameters were collected from 1,706 patients on admission. On day the second of hospitalization, venous fasting blood samples were routinely collected and analyzed for total blood count (TBC), total cholesterol, LDL cholesterol (LDL-C), HDL cholesterol (HDL-C), triglycerides (TG) and glucose (fasting plasma glucose; FPG).

Coronary angiography and PCI were performed according to the standard guidelines. During coronary angiography, infarct-related coronary artery was identified and hemodynamically significant atherosclerotic lesions in other coronary arteries were assessed. Atherosclerotic lesions narrowing the coronary artery lumen by at least 70% were considered hemodynamically significant.

Based on the results of laboratory tests and anthropometric measurements, the following indices were calculated:

- TyG index = ln [TG (mg/dl) x FPG (mg/dl)/2] [12]
- TyG-BMI = TyG index x BMI (kg/m^2^) [13]
- METS-IR = {ln [2 x FPG (mg/dl) + TG (mg/dl)] x BMI (kg/m^2^)}/ln [HDL-C (mg/dl)] [14]

We analyzed the prevalence of in-hospital death and MACCE (death from any cause, MI, stroke, revascularization) during the 12 months after STEMI and NSTEMI.

## Statistical analysis

Statistical analysis was performed using the STATISTICA 13.0 PL software (Tibco Software Inc, Palo Albo, CA, USA). Statistical significance was set at a p-value below 0.05. No data imputation was performed.

All tests were two-tailed. Nominal and ordinal data were expressed as percentages, while interval data as mean values ± standard deviation (SD). The distribution of variables was evaluated by the Shapiro–Wilk test. For comparison of data, the Student’s *t* test was used. Categorical variables were compared using χ2 test with correction for Yates’ continuity.

The influence of the factors on the prevalence of complications and death (in-hospital and long-term follow-up) was examined using logistic regression with the calculation of odds ratios (ORs). The cut-off values for TyG index, TyG-BMI, and METS-IR predicting in-hospital mortality and MACCE were determined using ROC curve analyses with the calculation of sensitivity and specificity for these values.

## Results

The study evaluated 1706 patients with STEMI and NSTEMI, including 680 with T2DM (39.8%) and 1026 without T2DM (60.2%). Coronary angiography was performed in all patients. In this group, a detailed description of the procedure was recorded in 1299 patients. In the remaining patients, PCI was performed but the data are missing. In the group of 1299 patients, 1293 patients underwent PCI. No significant atherosclerotic lesions that required coronary stent implantation were found in the remaining six patients. In 1706 patients, TyG index, Tyg-BMI and METS-IR indexes were assessed. The presence of MACCE was assessed within 12 months after acute coronary syndrome (ACS).

Baseline clinical characteristics group are given in Table 1. Patients with T2DM presented with more comorbidities and previously diagnosed CAD was more prevalent. The mean duration of T2DM was 8 ± 4 years.

**Table 1:** General characteristics of the study group (n = 1706)

Data	Total	Group 1 T2DM	Group 2 Non T2DM	p-value
	[N = 1706]	[N = 680]	[N = 1026]	
Age [years]	66 ± 11	70 ± 10	63 ± 12	< 0.001
Sex: Women N [%]	529 [31.0%]	255 [37.5%]	274 [26.7%]	< 0.001
Duration of diabetes [years]		8 ± 4		–
BMI [kg/m^2^]	28 ± 5	29 ± 5	27 ± 5	< 0.001
History of CAD N [%]	739 [43.3%]	417 [61.3%]	322 [31.4%]	< 0.001
History of myocardial infarction N [%]	549 [32.2%]	309 [45.4%]	240 [23.4%]	< 0.001
History of stroke N [%]	100 [5.9%]	57 [8.4%]	43 [4.2%]	< 0.001
AFib N [%]	169 [9.9%]	97 [14.3%]	72 [7.0%]	< 0.001
History of COPD N[%]	66 [3.9%]	31 [4.6%]	35 [3.4%]	0.282
History of chronic kidney disease N [%]	123 [7.2%]	85 [12.5%]	38 [3.7%]	< 0.001
History of chronic heart failure N [%]	242 [14.2%]	141 [20.7%]	101 [9.8%]	< 0.001
History of PCI N [%]	545 [31.9%]	304 [44.7%]	241 [23.5%]	< 0.001
History of CABG N [%]	201 [11.8%]	132 [19.4%]	69 [6.7%]	< 0.001
Hypertension N [%]	1161 [68.1%]	555 [81.6%]	606 [59.1%]	< 0.001
Hypercholesterolemia N [%]	556 [32.6%]	286 [42.1%]	270 [26.3%]	< 0.001
Smoking N [%]	410 [24.0%]	104 [15.3%]	306 [29.8%]	< 0.001
Left ventricular ejection fraction – (LVEF) [%]	42 ± 11	41 ± 12	43 ± 10	< 0.001

Data are presented as mean ± SD

(*BMI* body mass index, *CAD* coronary artery disease, *AMI* acute myocardial infarction, *AF* atrial fibrillation, *COPD* chronic obstructive pulmonary disease, *PCI* percutaneous coronary intervention, *CABG* coronary artery bypass grafting, *LVEF* left ventricular ejection fraction)

Table 2 shows laboratory characteristics used to assess the indices (TyG index, Tyg-BMI and METS-IR). Patients with T2DM had worse results of most laboratory tests, except for total cholesterol and LDL-cholesterol, which were lower in this group of patients.

**Table 2:** Laboratory characteristics

Data	Total	Group 1 T2DM	Group 2 Non T2DM	p-value
	[N = 1706]	[N = 680]	[N = 1026]	
Hemoglobin [mmol/L]	7.7 ± 1.4	7.5 ± 1.5	7.9 ± 1.4	< 0.001
Hematocrit [%]	36 ± 1	35 ± 1	37 ± 1	< 0.001
Erythrocytes [× 10^6^/μL]	4.1 ± 0.7	4.0 ± 0.7	4.1 ± 0.6	< 0.001
Leukocytes [× 10^3^/μL]	7.3 ± 2.3	7.1 ± 2.1	7.5 ± 2.4	< 0.001
Platelets [× 10^3^/μL]	198 ± 64	191 ± 65	204 ± 63	< 0.001
Total cholesterol [mg/dL]	198 ± 57	179 ± 53	210 ± 56	< 0.001
HDL-C [mg/dL]	52 ± 16	49 ± 15	53 ± 16	< 0.001
LDL-C [mg/dL]	121 ± 51	103 ± 48	132 ± 50	< 0.001
Triglycerides [mg/dL]	134 ± 79	138 ± 76	131 ± 81	0.001
Fasting glucose (mg/dL)	158 ± 72	190 ± 86	136 ± 50	< 0.001
Creatinine [mg/dL]	1.3 ± 0.8	1.4 ± 1.0	1.2 ± 0.8	< 0.001
eGFR CKD-EPI	64 ± 23	57 ± 23	69 ± 21	< 0.001
Max CK-MB level [ng/mL]	80 ± 104	61 ± 89	93 ± 112	< 0.001
Max troponin level [pg/mL]	312 ± 1391	186 ± 1106	394 ± 1544	0.007

Data are presented as mean ± SD

(HDL-C high-density lipoprotein cholesterol, *LDL-C* low-density lipoprotein cholesterol, *eGFR* CKD-EPI estimated glomerular filtration rate Chronic Kidney Disease Epidemiology Collaboration, *CK-MB* creatine kinase myocardial band isoenzyme)

Angiographic characteristics are presented in Table 3. Patients with T2DM had a higher prevalence of multivessel coronary artery disease (MVD) compared to those without T2DM. However, left anterior descending artery (LAD) was less frequently the infarct-related artery. Given the problems in identifying culprit vessels in NSTEMI patients, caution should be exercised when interpreting the data.

**Table 3:** Assessment of coronary angiography and PCI

Data	Total	Group 1 T2DM	Group 2 Non T2DM	p-value
	[N = 1299]	[N = 511]	[N = 788]	
Single-vessel coronary artery disease	426 [32.8%]	121 [23.7%]	305 [38.7%]	< 0.001
Two-vessel coronary artery disease	430 [33.1%]	178 [34.8%]	252 [32.0%]	0.314
Three-vessel coronary artery disease	341 [26.3%]	170 [33.3%]	171 [21.7%]	< 0.001
Left main disease	96 [7.4%]	51 [10.0%]	45 [5.7%]	0.006
Infarct related artery^a^				
RCA	450 [36.1%]	188 [38.4%]	262 [34.7%]	0.211
LAD	519 [41.7%]	185 [37.8%]	334 [44.2%]	0.030
Cx	255 [20.5%]	109 [22.2%]	146 [19.3%]	0.242
IM	22 [1.8%]	8 [1.6%]	14 [1.9%]	0.946
Number of stents implanted	1.9 ± 1.1	1.9 ± 1.0	1.9 ± 1.1	0.438
Number of dilated arteries	1.4 ± 0.6	1.4 ± 0.6	1.4 ± 0.7	0.959
Number of PCI during hospital stay	1.2 ± 0.5	1.3 ± 0.7	1.1 ± 0.4	< 0.001

Data are presented as mean ± SD

(*RCA* right coronary artery, *LAD* left anterior descending artery, *Cx* circumflex coronary artery, *IM* intermediate branch, *PCI* percutaneous coronary intervention)

^a^Given the problems in identifying culprit vessels in NSTEMI patients, caution should be exercised when interpreting the data

Table 4 shows the data on insulin resistance surrogates (TyG index, Tyg-BMI, METS-IR). Patients with T2DM had higher surrogate indices of insulin resistance (p < 0.001). Table 5 shows in-hospital and long-term prognosis. The rate in-hospital death was similar in the two groups. MACCE was more prevalent in patients with T2DM during 12 month follow-up (p < 0.001). Independent predictors of in-hospital death are presented in Table 6 and include age, female sex, decreased left ventricular ejection fraction, prior stroke, elevated TyG index, elevated creatinine levels and white blood count (WBC) and multivessel CAD.

**Table 4:** Surrogate indices of insulin resistance

Data	Total	Group 1 T2DM	Group 2 Non T2DM	p-value
	[N = 1706]	[N = 680]	[N = 1026]	
TyG index	9.0 ± 0.7	9.3 ± 0.7	8.9 ± 0.7	< 0.001
TyG-BMI	255 ± 52	271 ± 51	245 ± 49	< 0.001
METS-IR	2.4 ± 0.3	2.5 ± 0.3	2.4 ± 0.2	< 0.001

Data are presented as mean ± SD

(*TyG index* triglyceride-glucose index, *TyG-BMI* triglyceride-glucose body mass index, *METS-IR* metabolic score for insulin resistance)

**Table 5:** In-hospital and long term outcomes

Data	Total	Group 1 T2DM	Group 2 non T2DM	p-value
	[N = 1706 %]	[N = 680 %]	[N = 1026 %]	
In-hospital mortality N [%]	58 [3.4]	29 [4.3]	29 [2.8]	0.142
MACCE N [%]	292 [18.9]	159 [25.8]	133 [14.4]	< 0.001
All-cause mortality N [%]	165 [10.6]	91 [14.7]	74 [8.0]	< 0.001
Myocardial infarction N [%]	113 [7.3]	66 [10.7]	47 [5.1]	< 0.001
Stroke N [%]	26 [1.7]	14 [2.3]	12 [1.3]	0.211
Revascularization N[%]	112 [7.3]	64 [10.4]	48 [5.2]	< 0.001

Data are presented as mean ± SD

(*MACCE* major adverse cardiac and cerebrovascular event)

**Table 6:** Results of the multivariate logistic regression for factors influencing the occurrence of in-hospital death

	OR	− 95% Cl	+ 95% Cl	p-value
Age (per 10 year increment)	1.66	1.02	2.69	0.041
Female sex	4.91	1.79	13.51	0.002
LVEF (per 5% decrement)	1.65	1.34	2.05	< 0.001
Prior stroke	4.59	1.23	17.14	0.024
Creatinine (per 1 mg/dL increment)	1.68	1.25	2.25	< 0.001
WBC (per 10^3^/μL increment)	1.63	1.33	2.00	< 0.001
TyG index (1 unit increment)	2.48	1.28	4.80	0.007
Three-vessel CAD	8.03	1.52	42.61	0.014

(*LVEF* left ventricular ejection fraction, *WBC* white blood cells, *TyG* index triglyceride-glucose index, *CAD* coronary artery disease)

Fasting glucose concentration and the glucose concentration on admission are not independent factors of in-hospital deaths. Independent predictors of MACCE during 12-month follow-up are given Table 7—these include age, decreased left ventricular ejection fraction, prior CAD, FPG, WBC, and cholesterol-LDL.

**Table 7:** Results of the multivariate logistic regression for factors influencing the occurrence of MACCE

	OR	− 95% Cl	+ 95% Cl	p
Age (per 10 year increment)	1.23	1.07	1.41	0.005
LVEF (per 5% decrement)	1.17	1.10	1.25	< 0.001
Prior CAD	1.57	1.16	2.12	0.004
Glucose (per 50 mg/dL increment)	1.19	1.08	1.31	< 0.001
HGB (per 1 mmol/L increment)	1.31	1.16	1.48	< 0.001
LDL-C (per 50 mg/dL increment)	1.19	1.02	1.40	0.031

(*LVEF* left ventricular ejection fraction, *CAD* coronary artery disease, *HGB* Hemoglobin, *LDL-C* low-density lipoprotein cholesterol)

**Fig. 1:**
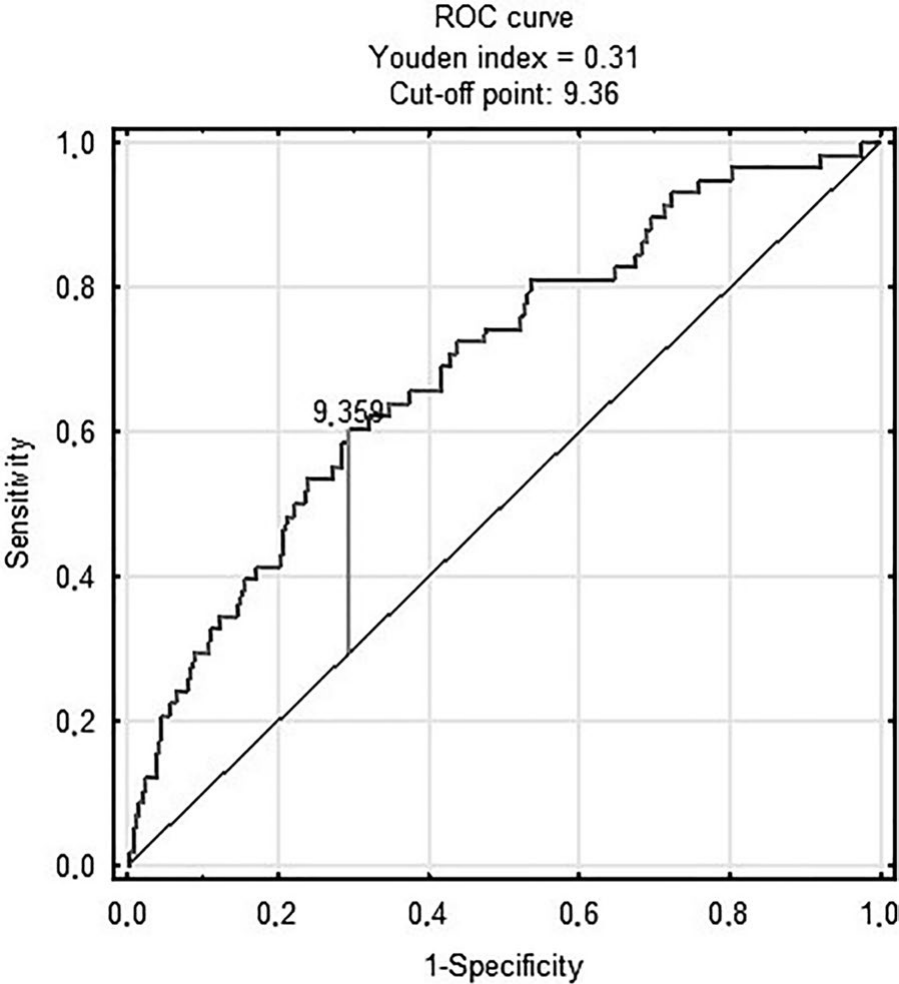
TyG index and in-hospital death

**Fig. 2:**
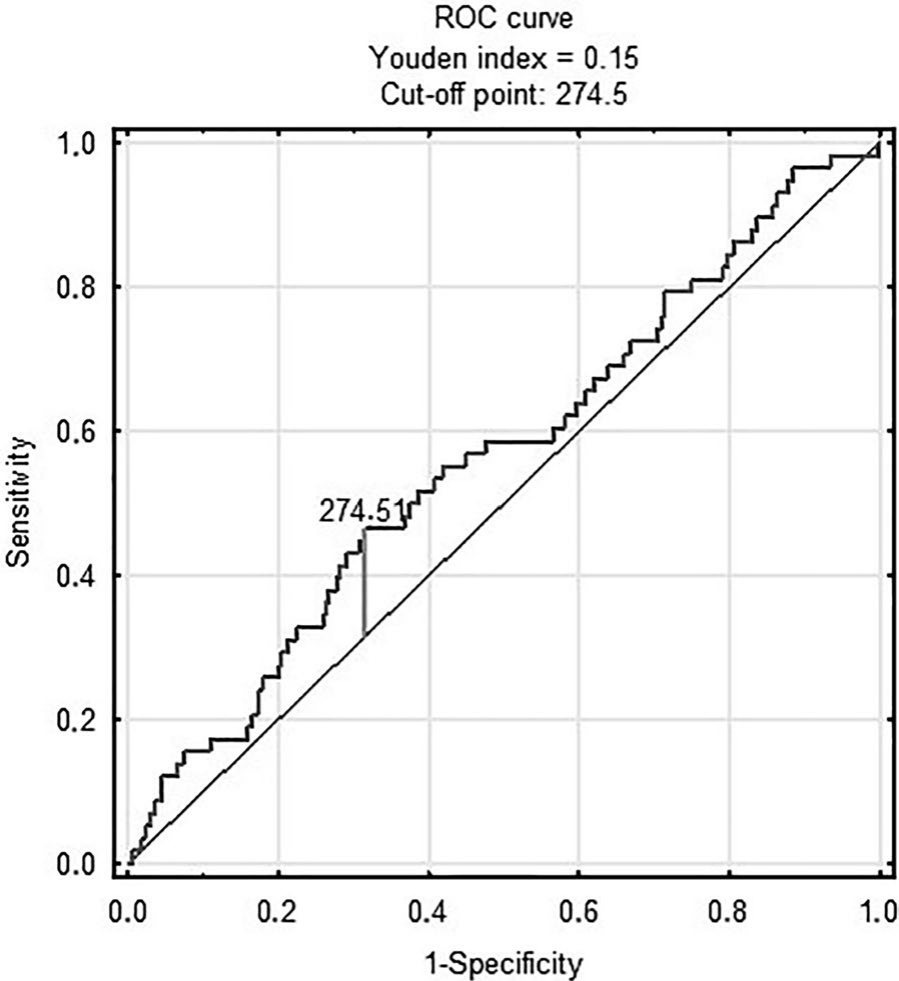
TyG-BMI and in-hospital death

Figure 1 shows ROC analysis of the TyG index in predicting in-hospital death [AUC 0.69 (95% CI 0.62–0.76); p < 0.001 for the cut-off point 9.36 (sensitivity = 0.6, specificity = 0.71)]. There was no prognostic value of TyG-BMI in predicting in-hospital death (Fig. 2)

**Fig. 3:**
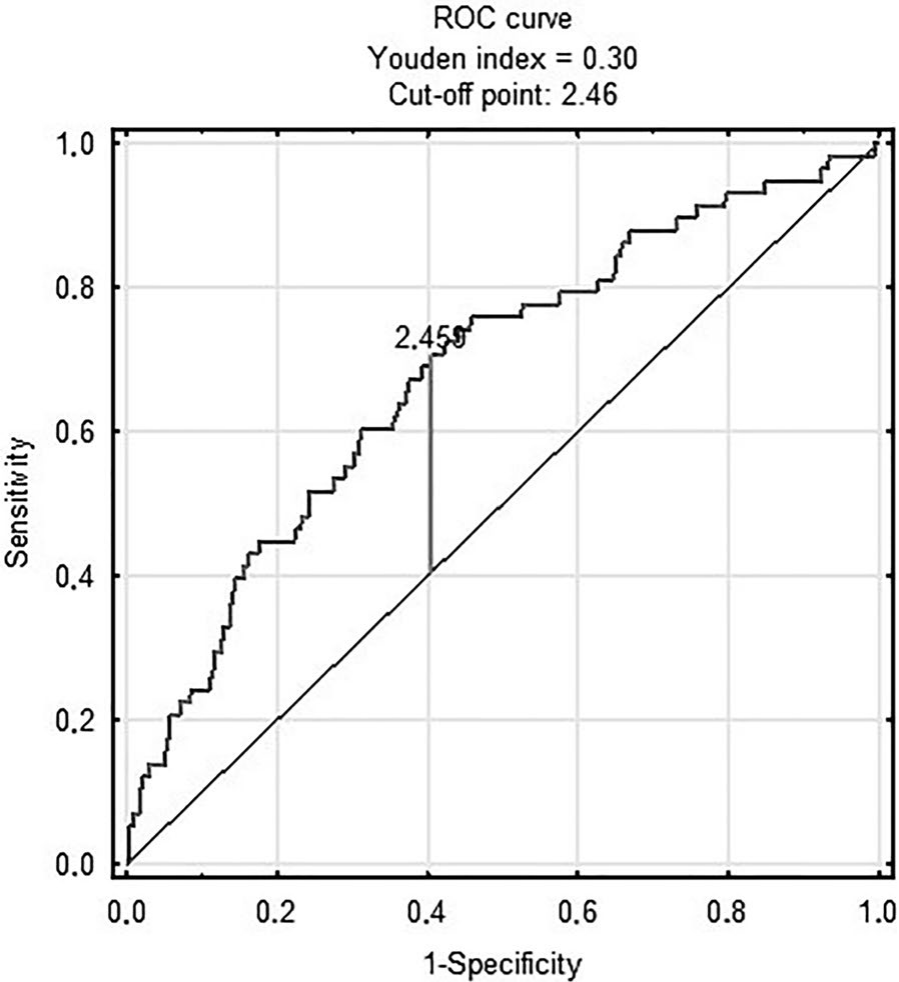
METS-IR and in-hospital death

**Fig. 4:**
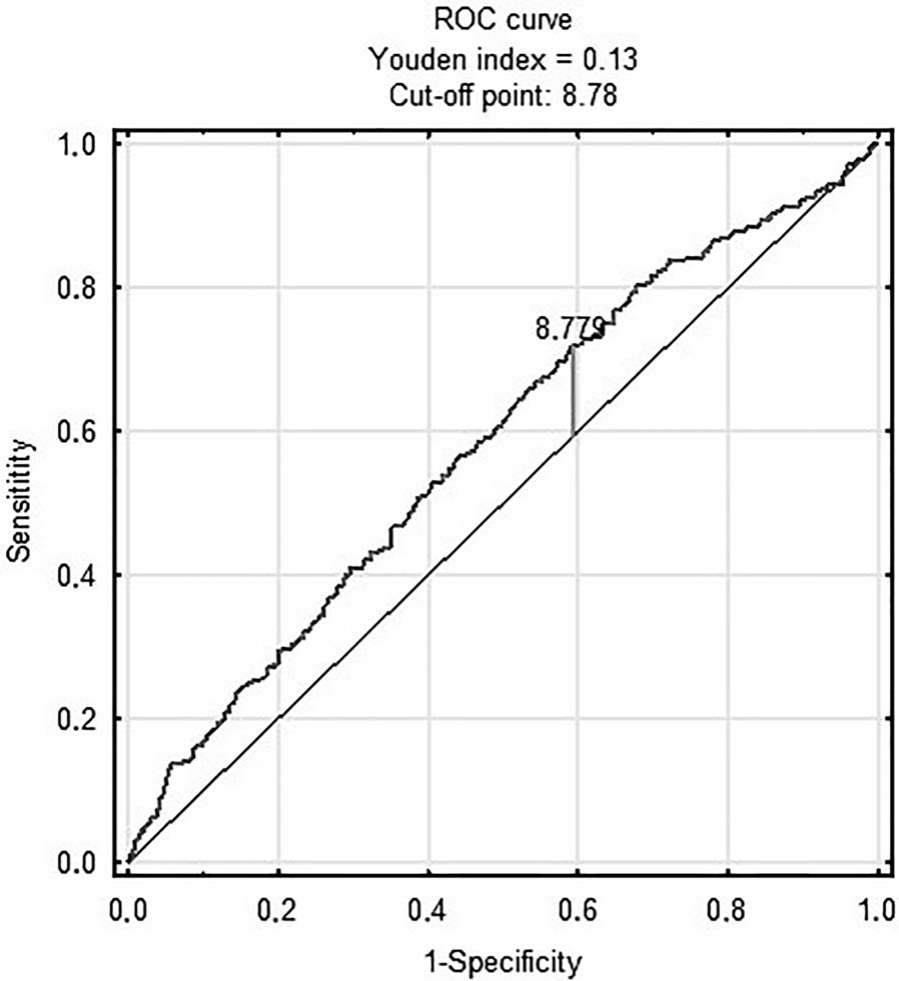
TyG index and MACCE

**Fig. 5:**
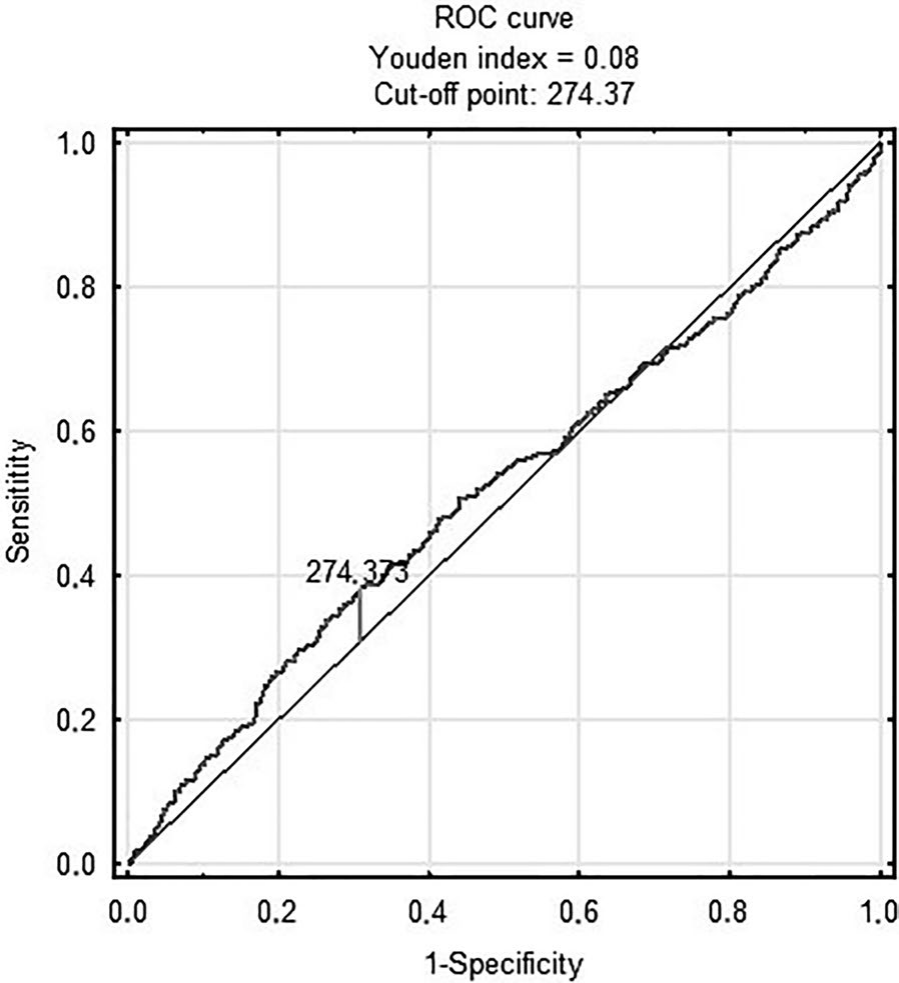
TyG-BMI and MACCE

**Fig. 6:**
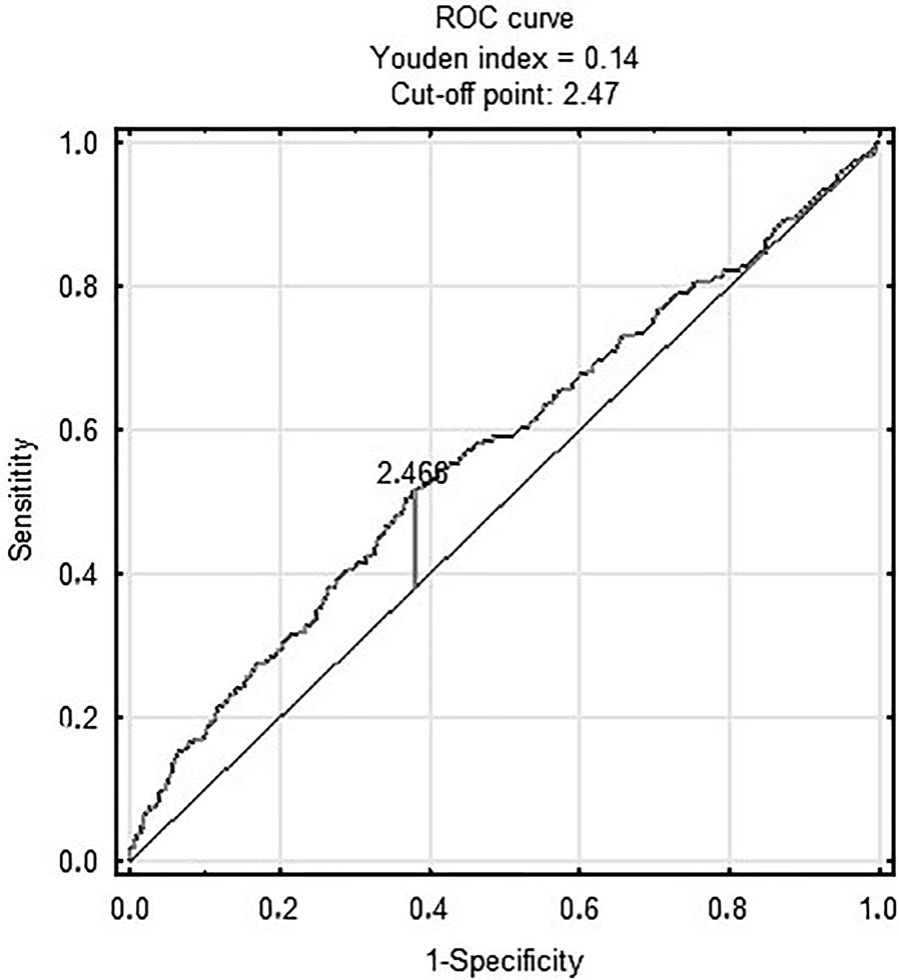
METS-IR and MACCE

Figure 3 shows prognostic value of METS-IR in predicting in-hospital death [AUC 0.68 (95% CI 0.61–0.75); p < 0.001 for the cut-off point 2.46 (sensitivity = 0.71, specificity = 0.6)]. Figure 4 shows prognostic value of the TyG index in predicting MACCE [AUC 0.58 (95% CI 0.55–0.62); p < 0.001 for the cut-off point 8.78 (sensitivity = 0.7, specificity = 0.41)]. There was no predictive value of TyG-BMI for MACCE (Figs. 5). Figure 6 shows prognostic value of METS-IR in predicting MACCE [AUC 0.57 (95% CI 0.53–0.61); p < 0.001 for the cut-off point 2.47 (sensitivity = 0.52, specificity = 0.72)].

## Discussion

Our study is the first to use all three indices (TyG index, TyG-BMI and METS-IR) to estimate the risk of in-hospital death and long-term MACCE in patients with and without T2DM after STEMI or NSTEMI. This single-center retrospective study showed that an increase in the TyG index was an independent risk factor for in-hospital death in patients with STEMI and NSTEMI. None of the three indices were an independent risk factor for MACCE during 12 month follow-up. TyG index and METS-IR indices had low predictive value in predicting the occurrence of MACCE within 12 months after STEMI and NSTEMI. TyG-BMI had no predicting value for in-hospital death or MACCE.

Analyzing the groups of patients with and without T2DM, we showed that patients with MI and T2DM had a significantly worse prognosis due to a higher prevalence of MACCE and its components including death, MI, or repeat revascularization, which is in line with most studies [15–18]. The three indices (TyG index, TyG-BMI, METS-IR) were used as surrogates for insulin resistance, which can be an indirect indicator of the metabolic syndrome. The TyG index was shown to predict metabolic syndrome better than the HOMA-IR [19]. Chenge et al. showed that TyG-BMI and METS-IR were independent risk factors for hypertension, had a good predictive value for the prevalence of hypertension, and TyG-BMI was superior to METS-IR in this respect [20]. In their study on patients with T2DM, Han et al. showed a positive correlation between TyG index, TyG-BMI, METS-IR and TG/HDL-cholesterol and hyperuricemia. Of the four surrogates of insulin resistance, TG/HDL-c was the best marker for identifying hyperuricemia in patients with T2DM [21]. It is known that insulin resistance influences not only metabolic disorders, such as dyslipidemia, obesity, and disorders of carbohydrate metabolism but also atherosclerosis and the progression of CVD [22]. Therefore, we attempted to use surrogate markers of insulin resistance that are easy to calculate for prognostic purposes for in-hospital death and MACCE in patients after STEMI or NSTEMI.

Our results are in agreement with those of Gou et al. who showed that the TyG index and TG/HDL-Cholesterol were significant predictors of in-hospital mortality in non-diabetic acute MI patients [23]. In our study, multivariate logistic regression showed that TyG index was an independent risk factor for in-hospital death in patients with STEMI and NSTEMI. Per one unit increment in the TyG index, the risk of in-hospital death increased 2.5-fold. In-hospital death was also assessed by Liao et al. in a group of patients hospitalized in an intensive care unit (ICU). Patients were hospitalized for various conditions (not only due to MI). Liao et al. showed that the TyG index was strongly associated with increased mortality from any cause in patients in the ICU. Therefore, it may be useful for identifying critically ill patients at high risk of death [24]. Another study found that a higher TyG index was associated with a higher risk of in-hospital mortality in patients with ischemic stroke [25]. It seems that the TyG index is a good predictor of in-hospital death not only in the group of post-myocardial infarction patients (as we showed) but also in those with severe general condition due to other diseases.

The TyG index was also assessed as a predictor of MACCE in a group of 1,092 STEMI patients who underwent PCI. The prevalence of MACCE and mortality from any cause at 30 days, 6 months and 1 year after PCI was higher in STEMI patients with TyG index levels in the highest quartile. The area under the curve (AUC) of the TyG index predicting the occurrence of MACCE in STEMI patients after PCI was 0.685 (95% CI 0.610–0.761; p = 0.001) [26]. In our study, TyG index prognostic value for MACCE was 0.582 (95% CI 0.546–0.619; p < 0.001). Although the results were comparable, the predictive value was low for using these indices for prognostic purposes.

METS-IR was evaluated in patients with ischemic cardiomyopathy (ICM) and T2DM. The index was predictive of the occurrence of MACE, regardless of known cardiovascular risk factors. These results suggest that METS-IR may be a useful marker for MACE risk stratification in patients with ICM and T2DM [27]. Our results pertaining to METS-IR are similar to those obtained by Drwiła-Stec et al. In their study, MACE and all-cause mortality at 1-year follow-up occurred in 7.9% of patients in the STEMI group and 10.9% in the NSTEMI group. Neither of the indexes (METS-IR and TyG-BMI) was a predictor of MACE in the STEMI or NSTEMI groups [28]. It seems that currently METS-IR cannot be considered a good predictor of MACCE after MI.

We demonstrated that METS-IR could potentially be used as a prognostic factor for in-hospital death. However, the sensitivity (70%) and specificity (60%) were insufficient and the predictive value was low. Therefore, further studies are warranted to consider the METS-IR index an important predictor of in-hospital death.

We are the first to assess TyG-BMI for predicting in-hospital death and MACCE. We showed that TyG-BMI was not suitable for the predicting in-hospital death or long-term MACCE. Studies confirmed that TyG-BMI is significantly associated with the severity of CAD and is an independent risk factor for multivessel CAD [3]. Zhang et al. found that TyG index, TyG-BMI, TG/HDL-Cholesterol and METS-IR could be valuable predictors of CAD severity. Of the four indices above, METS-IR had the highest predictive value for CAD severity, followed by TyG-BMI [3]. Apart from our study, Cheng et al. also evaluated TyG-BMI as a predictor of MACCE in patients undergoing PCI [29]. Higher TyG-BMI was proportional to the increased prevalence of MACCE in female and elderly patients. However, including TyG-BMI did not improve risk prediction over traditional risk factors in elderly or female patients [29].

Although the above-mentioned indices have been effective in estimating insulin resistance, some of them do not seem to have prognostic significance for in-hospital death and long-term MACCE. Only the TyG index was identified as an independent risk factor for in-hospital death in patients with STEMI and NSTEMI. Confirmation of this finding in subsequent clinical studies may lead to the integration of TyG index assessment upon hospital admission and the adoption of therapeutic approaches different from those used thus far (potentially SGLT-2 inhibitors or GLP-1 receptor agonists or amylin hold promise in patient with ACS) [30–32]. Considering the use of cardioprotective drugs, such as SGLT-2 inhibitors or GLP-1 receptor agonists, in patients with and without diabetes, it is valuable to evaluate their impact on the TyG index and potential benefits in reducing in-hospital mortality and MACCE after STEMI and NSTEMI [30, 31]. Given the study period we were unable to analyze the effects of SGLT-2 inhibitors or GLP-1 receptor agonists in our population.

The main strength of this study includes a large sample size. The study is the first to use all three novel markers of insulin resistance and progression of atherosclerosis (TyG index, TyG-BMI and METS-IR) at once to estimate prognosis after AMI. Nevertheless, this study should be viewed in light of its limitations. First, this is single-center, retrospective and an observational study, thus the results could indicate association but not causation. Therefore, the results of our study should be confirmed in a multicenter, prospective study. In addition, the study was conducted between 2013 and 2021, during which SGLT-2 inhibitors or GLP-1 receptor agonists were not reimbursed and rarely used. These drugs potentially might have influenced the results of prognostic value of the novel biomarkers.

## Conclusions

In conclusion, of the insulin resistance surrogates evaluated in the study, only TyG index was an independent risk factor for in-hospital death in patients with STEMI or NSTEMI. None of the insulin resistance surrogates were an independent risk factor for MACCE during the 12-month follow-up. TyG index and METS-IR have a low predictive value for predicting MACCE within 12 months after STEMI or NSTEMI.
